# Phage display derived peptides for Alzheimer's disease therapy and diagnosis

**DOI:** 10.7150/thno.68636

**Published:** 2022-01-31

**Authors:** Xiancheng Zhang, Xiaoyu Zhang, Huiling Gao, Guangyan Qing

**Affiliations:** 1 Key Laboratory of Separation Science for Analytical Chemistry, Dalian Institute of Chemical Physics, Chinese Academy of Sciences, 457 Zhongshan Road, Dalian 116023, P. R. China.; 2 College of Life and Health Sciences, Northeastern University, 3-11 Wenhua Road, Shenyang, 110819, P. R. China.; 3 Division of Biotechnology, Dalian Institute of Chemical Physics, Chinese Academy of Sciences, 457 Zhongshan Road, Dalian, 116023, P. R. China.; 4 College of Chemistry and Chemical Engineering, Wuhan Textile University, 1 Sunshine Road, Wuhan 430200, P. R. China.

**Keywords:** Alzheimer's disease, Phage display, Affinity, Peptide therapy, Brain target, Early detection

## Abstract

Alzheimer's disease (AD) is an incurable and fatal progressive neurodegenerative disorder associated with memory and cognition impairment. AD is one of the top medical care concerns across the world with a projected economic burden of $2 trillion by 2030. To date, however, there remains no effective disease-modifying therapy available. It is more important than ever to reveal novel therapeutic approaches. Peptide-based biotherapeutics has been a great potential strategy attributed to their distinct and superior biochemical characteristics, such as reproducible chemical synthesis and modification, rapid cell and tissue permeability, and fast blood clearance. Phage display, one of today's most powerful platforms, allows selection and identification of suitable peptide drug candidates with high affinities and specificity toward target, demonstrating the potential to overcome challenges and limitations in AD diagnosis/treatment. We aim to provide the first comprehensive review to summarize the status in this research direction. The biological overview of phage display is described, including basic biology of the phage vectors and construction principle of phage library, biopanning procedure, mirror image phage display, and various binding affinity evaluation approaches. Further, the applications of phage display in AD therapy, targeted drug delivery, and early detection are presented. Finally, we discuss the current challenges and offer a future outlook for further advancing the potential application of phage display on AD and other neurodegenerative diseases.

## Introduction

Alzheimer's disease (AD), the most prevalent cause of dementia, is a heterogeneous and progressively neurodegenerative disease, most of which occur in the mid to late adulthood 1. AD is clinically characterized by memory impairment, aphasia, apraxia, agnosia, impairment of visual spatial skills, executive dysfunction, and bedridden in the final stage 2. Worldwide, over 50 million people currently suffer from dementia, and the number of affected individuals is expected to be 152 million in 2050, of which about 60% to 70% are AD patients 3. It is estimated that the costs associated with social dementia will rise to $2 trillion globally by 2030. AD has been one of the great health-care challenges in the 21st century with a tremendous medical and socioeconomic burden worldwide.

To date, several biological molecules are considered to be the main reasons for AD, and a series of hypotheses have emerged to explain the AD pathogenesis, mainly including amyloid cascade hypothesis, tau protein hypothesis, metal ion disorder hypothesis, oxidative stress hypothesis, and cholinergic hypothesis, *etc.* 4-6. Among them, extracellular senile plaques (SPs) formed by the deposition of Aβ from amyloid precursor proteins sequentially cleaved by multiple splicing enzymes, and neurofibrillary tangles (NFTs) contributed by the aggregation of intracellular hyperphosphorylated tau proteins are two prominent histopathological hallmarks of AD 7. The imbalance of metal homeostasis in the brains of AD patients is also of great concern 5. Excessive metal ions accumulated in brain, such as copper, zinc, and iron ions, can coordinate with Aβ, promoting the formation of neurotoxic Aβ oligomers and producing oxidative stress molecules. These factors damage intracellular biological macromolecules (*e.g.* lipid, protein and nucleic acids), which cause neural dysfunction and finally lead to neuronal death 8.

Despite it has been more than 100 years since German doctor Alois Alzheimer first discovered AD 9, there remains no effective disease-modifying therapy available today. The currently four Food and Drug Administration (FDA) approved therapeutic drugs for symptomatic AD can only ameliorate modest cognitive impairment and dysfunction, and most small molecule drugs and monoclonal antibodies in clinical trials targeting Aβ production, aggregation, and clearance have not demonstrated any efficacy in slowing down cognitive decline or improving overall function. The recent FDA accelerated approval of aducanumab (Aduhelm) for the treatment of AD is still controversial 10. These many trial failures highlight the need for new approaches and strategies for the AD therapy design.

Over the recent decade, peptide biotherapeutics has been an attractive approach. There are more than 60 FDA-approved peptide drugs on the market, and more than 400 therapeutic peptides are currently in clinical development 11. In comparison to small molecules, such as proteins and antibodies, peptides represent a unique class of pharmaceutical compounds attributed to their distinct biochemical characteristics, such as reproducible chemical synthesis and modification, rapid cell and tissue permeability, fast blood clearance and low immunogenicity. These robust properties make them appealing candidates for AD applications, such as molecular imaging, disease diagnosis, and therapy.

Peptides are usually composed of short amino acid chains (less than 50 AA) without complex spatial structure. Their function is primarily determined by the amino acid sequences, therefore, how to design and screen peptide sequences is crucial to their functionality. Over the past few years, a number of strategies have been designed to screen specific amino acid sequences against several target molecules 12, 13. Among them, high-throughput screening (HTS) techniques such as phage, mRNA/DNA, yeast, ribosomal, and cell display technologies facilitate the rapid identification of new peptide lead, with phage display being the best understood and most commonly used. Phage display has proved to be predominant platforms for discovery of suitable peptide ligand, including disease-specific or organ-specific peptides 14, cancer cell/tumor targeting peptides 15, immune checkpoint antagonists 16, and biosensors 17, *etc*.

**Figure 1:**
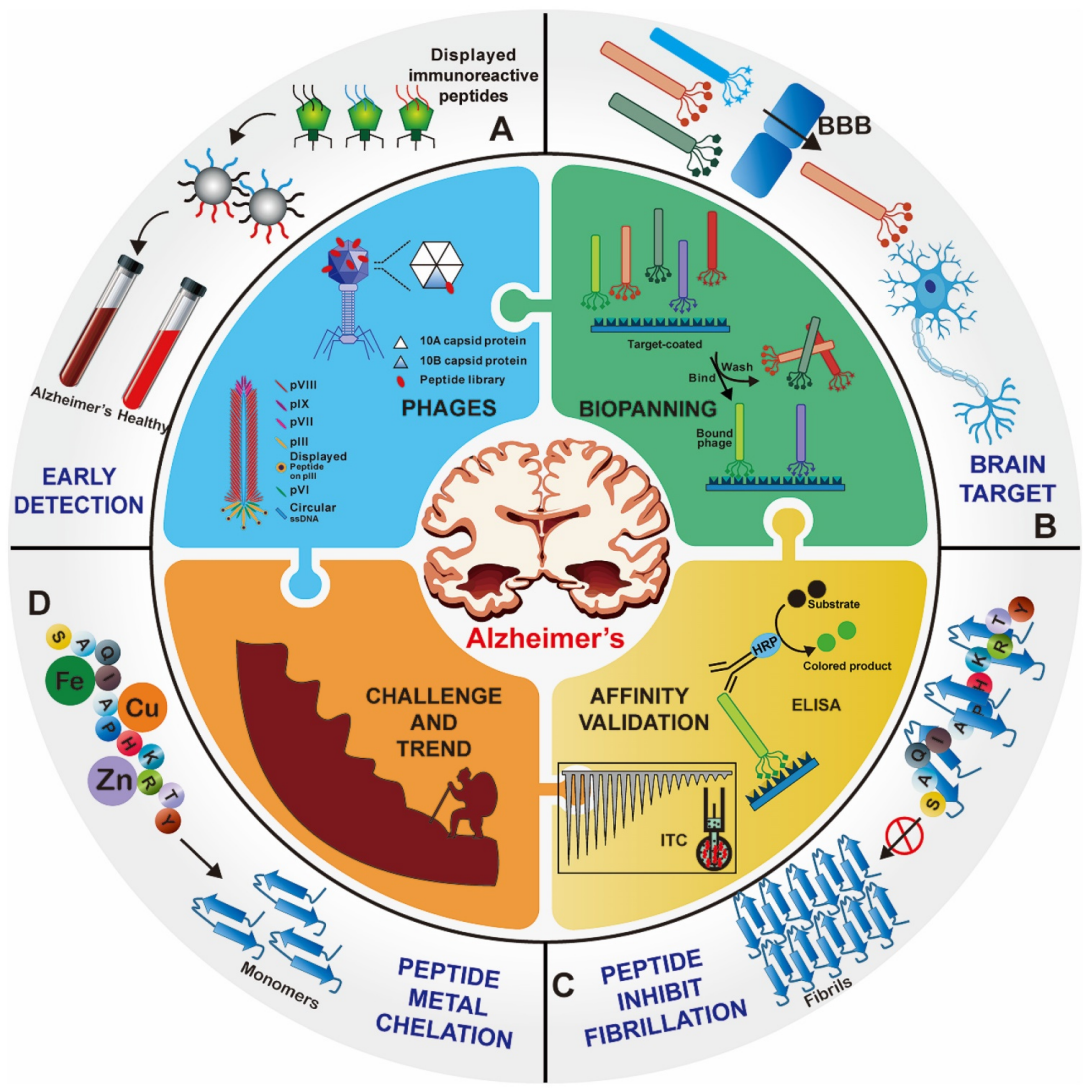
**Overview of phage display technology for Alzheimer's disease applications.** Foreign peptides are displayed on the capsid protein of phage vectors. Through repeated biopanning against target, target-specific phages are selected. Various peptides screened from the phage display technology have been successfully applied for the early detection (A), brain targeted drug delivery (B), and treatment (C) and (D) of AD.

Over the past decade, great efforts have been made to develop peptide agents for neurodegenerative diseases, particularly against AD, through phage display strategies. Here we would like to provide the first comprehensive review of the up-to-date research of phage display technique in AD applications. For better understanding, the elementary biological overview of phage display technology, including phage vectors species, selection procedure, mirror image phage display and various affinity evaluation methods, is introduced. Then the applications of phage display in AD therapy, brain drug delivery and early detection are highlight presented. Finally, we focus on the existing bottlenecks and challenges in the application of phage display in AD and other neurodegenerative diseases, as well as future trends and directions **(Figure 1)**.

## Overview of phage display

The field of phage display originated from the initial discovery by George Smith in 1985 18. Exogenous peptide was fused into the phage coat protein and displayed on the surface to form combinatorial phage. Phage display establishes physical linkage between peptide/protein and DNA sequence, which allows for rapid separation based on binding affinity with a specific target molecule and facilitates characterization of displayed peptide/proteins after selecting phages with desired binding properties 17, 19. Greg Winter and his colleagues applied this technology to therapeutic protein engineering, especially in the discovery and preparation of antibodies. For their outstanding contributions to “directed evolution” in the field of biochemistry, Smith and Winter both won a quarter share of the 2018 Nobel Prize in Chemistry 15.

### Biology of the phage vectors

**Figure 2:**
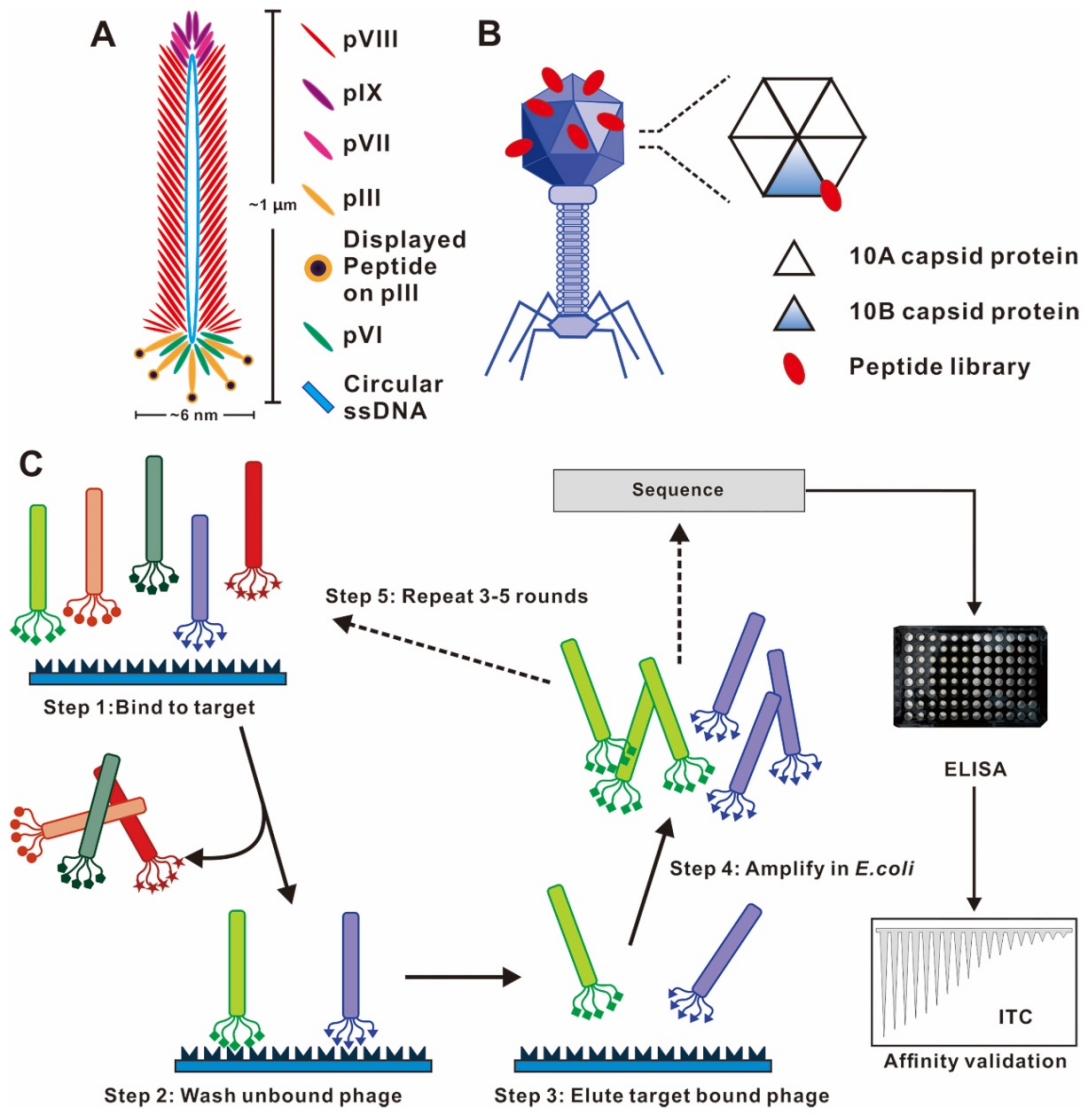
**Phage vector structures and the general biopanning procedure.** (A) Schematic representation of M13 phage. M13 phage DNA genome surrounded by five capsid proteins. pIII and pVIII capsid proteins are usually utilized in inserting foreign peptide. (B) Representative T7 lytic phage structure. The T7 phage head is composed of 415 copies of the capsid protein 10A and 10B. The genetically engineered peptide sequence can be modified on the capsid 10B. (C) Biopanning process of phage-displayed peptide libraries. The phage library is bound to the target molecular. The weakly bound and unbound phages are removed by stringent washes, and phage associated with the target are eluted and amplified in *E. coli*. Three-five rounds of biopanning are necessary, and then the phage genome is sequenced. (A) Reprinted with permission from 26. Copyright 2017 American Chemical Society. (B) Reprinted with permission from 25. Copyright 2014 American Chemical Society. (C) Reprinted from www.neb.com (2021) with permission from New England Biolabs.

Phages are viruses that infect bacteria. The most commonly used phage vectors for phage display are a member of the filamentous family (M13, Fd, f1). Wild-type M13 phage infection of enteric (gut) bacterium *Escherichia coli* is a nonlytic process without killing the host cell and replicates by assembling in the bacterial periplasm and secreting through the outer membrane. It has a rod-shaped structure with a length of 1 μm and a width of 6 nm (**Figure 2A**). The structure of the M13 phage is relatively simple, which comprises of a single-stranded, circular DNA genome surrounded by its correspondingly encoded protein outer shell ~ termed capsid. The capsid consists of a long tubular array of ∼2700 copies of one major coat protein (pVIII) subunits, and is capped at each end by 5 copies each of four minor proteins (pIII and pVI on one tip and pVII and pIX on the other tip) 20. pIII and pVIII capsid are the two commonly used coat proteins due to their wider tolerance range or stronger productivity 21. The M13 phage display system is in a position to display folded proteins carrying disulfide bonds 22. Numerous enzymes, functional antibody fragments, and inhibitors have been displayed using the M13 system 23. Another representative phage vector is the T7 phage species, which has an icosahedral head and a short tail (**Figure 2B**) 20, 24. The outer shell of the T7 phage head is composed of the 10A and 10B capsid proteins, and exogenous peptide sequences are typically displayed as C-terminal fusions of the 10B capsid protein to avoid the stop codon problem 25. The T7 phage is lytic, so display and reproduction are not dependent on the secretion through the bacterial membrane 13.

The construction of the phage library requires the genetic engineering method to insert massive, random, exogenous oligonucleotide fragments into the structural genes of the phage to realize its transcription and translation, and the corresponding foreign peptide/protein encoded by the foreign gene will be displayed on the specific sites of phage capsid proteins. The phage library represents a mixture of millions of phage particles, each displaying a unique and random set of peptide/protein 26. According to the size of displayed peptide, epitope or antibody and the nature of antigen, different phage libraries have been constructed, mainly divided into two types of libraries, phage antibody libraries and phage peptide libraries. Phage display of single-chain V-domain antibody fragments (scFv), a fragment of antigen binding (Fab), and fully human monoclonal antibodies (mAbs) belong to the phage antibody library, which requires small size, easy cloning, and large-capacity phage vectors load 27, 28. Random phage peptide libraries are now one of the most extensively used types of phage display constructs, which provides a basis for selection affinity peptides with specific target molecules. The linear random peptide library can be constructed through introducing degenerate oligonucleotides into the phage genomes. Currently, peptides ranging in length from 6 to 43 amino acids have been successfully displayed as peptide-capsid fusion proteins on phages 29. In 2005, Scholle *et al*. generated 22 different phage peptide libraries ranging from 8-20 amino acids in length with sizes of up to 10^11^ clones, and have used these libraries to select peptide ligands to several protein targets 30, 31. Excepted homemade libraries, commercial random peptide libraries and commercial vectors, such as PhD series phage libraries from New England Biolabs, are available to accelerate the work of scientific researchers 32.

### Phage display biopanning procedure

Through an evolutionary selection process called biopanning, functional peptides with high affinity and specificity toward specific targets could be selected from a large random library containing up 10^9^ phage clones. The biopanning procedures generally include the following steps (**Figure 2C**). First, a customized phage library could be constructed to display desired foreign peptides. Second, the phage library is incubated with the target molecule (*e.g.*, peptide, protein and cell) to achieve binding. Billions of phages with randomly displayed peptides competitively bind to the target molecules, and potential peptides with stronger affinities are preferentially retained. Third, the weakly bound and unbound phages were removed by a wash buffer. Fourth, a low pH buffer or competitive elution is used to elute the high target-bound phages, and the eluted bound phages infect host bacteria to become a more selective phage library for the next round of biopanning. Three to five rounds of biopanning are indispensable for obtaining the phages with high affinities for the target molecule. In each round, the stringency and effectiveness of selection are improved by increasing the number of washing and reducing the amount of target molecules. Special care should be taken to avoid wild-type phage contamination during the biopanning procedure, as even vanishingly small levels of contamination can result in a majority of phage pool becoming wild-type phages after three rounds of biopanning. Subsequently, the foreign DNA inserted in the phage genome is sequenced after a final round of selection. The encoded amino acid sequences are the peptide ligand that binds to the target molecule, and the specificity verification of ligand was required in subsequent tests.

**Figure 3:**
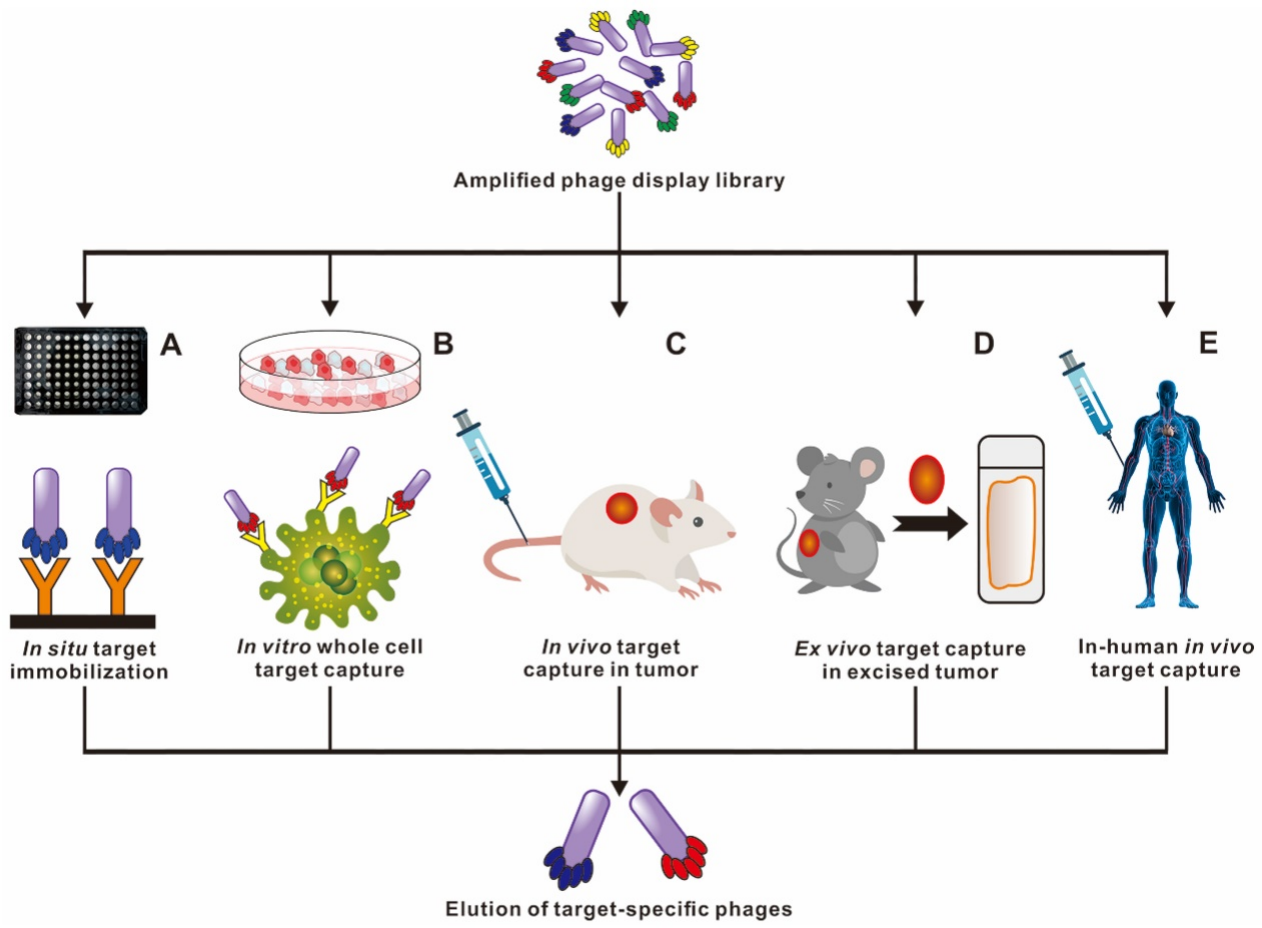
** Ample strategies in capturing high affinity and targeted peptide through phage display selection.** Multiple phage biopanning approaches to cater for specific needs of each experiment. Licensed under a Creative Commons Attribution (CC BY) license.

Phage selection can be carried out under different environmental conditions to isolate specific and desired functional peptides (**Figure 3**). *In situ* phage selection is the simplest and most versatile method, requiring only the coating of the target molecule to the surface of the well plate or bead, and the entire selection is performed without a living system. For example, the selection of Aβ in the current literature used biotin-conjugated Aβ, that was fixed in the well plate pre-coated with streptavidin to carry out multiple rounds of biopanning 33. Larbanoix *et al*. also described the Aβ immobilization on the bottom of the polystyrene well plate by hydrophobic absorption. 34, 35 Ample researches for selection functional peptides are also being conducted in cells, *in vivo*, *ex vivo* and even in patients to better mimic cellular and body original conditions. Phage *in vitro* cell selection provides a method for peptides recognition and binding specifically to individual cells.* Majerova* et al. used a 12-mer phage library to screen against the mouse blood-brain barrier (BBB) cell model, two BBB shuttle peptides were obtained capable of effectively permeating the brain, which could serve as transporters for brain drug delivery 36. It is common knowledge that the *in vitro* condition is quite different from the complex* in vivo* environment. Phage biopanning in living animals and even in patients is aimed at producing organ/tissue targeted peptides that are closer to the physiological conditions. Not the same as *in vitro* selection, the *in vivo* selection procedure involves systemic intravenous injecting the phage library into the body, and after a period of circulation, the desired organ/tissue is collected, homogenized and the phage extracted for sequencing 37, 38. Arap *et al*. reported the first in human phage display selection to identify several peptides that home to specific vascular beds in 2002 39. A patient received an intravenous infusion of a phage peptide library and underwent tissue biopsies of several organs 15 min later, followed by bioinformatical analysis of thousands of phage clones. They reported a peptide with the sequence of CGRRAGGSC, which could specifically home to prostate vasculature rather than other organs. It is worth noting that the biosafety of phages *in vivo* application should be taken seriously.

### Mirror image phage display

**Figure 4:**
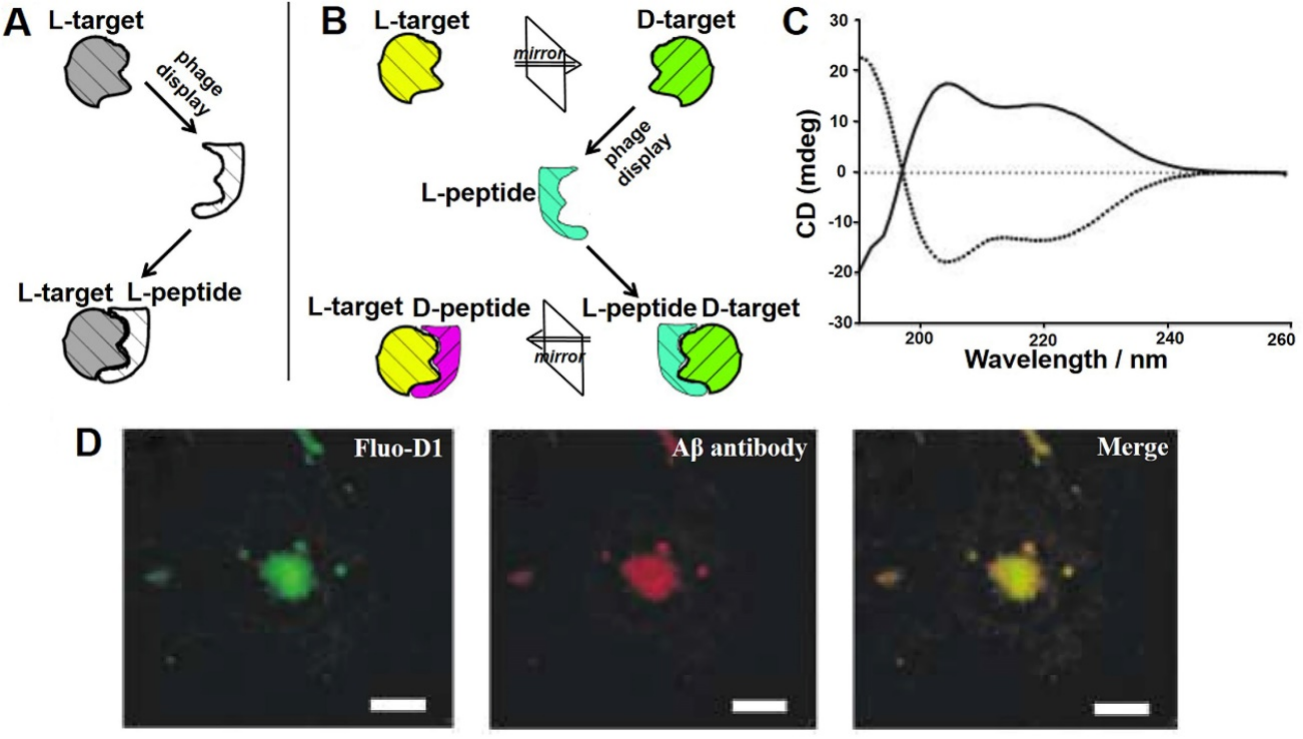
**Mirror image phage display and its application in AD.** Schematic representation of the principles of (A) common phage display and (B) mirror-image phage display. (A) A phage displayed peptide library is screened for L-peptide that bind to a given target, which consists of L-amino acids (L-target). (B) A phage displayed peptide library is screened for L-peptide that bind to a given target, which consists of D-amino acids (D-target). The peptide composed of D-amino acids (D-peptides) with the same L-peptide sequence could bind to naturally occurring L-target. (C) CD analysis showed that biotin-L-Aβ (1-42) (dotted line) and biotin-D-Aβ-(1-42) (solid line) had mirror symmetry. The Aβ composed of all-D amino acid residues was prepared as the target for phage selection. (D) Immunofluorescence staining of brain sections of AD patient with fluorescently labeled D1 (Fluo-D1) and commercially Aβ antibody with a Cy3-labeled secondary antibody. Reprinted with permission from 40, 43. Copyright 2003 WILEY-VCH Verlag GmbH & Co. KGaA, Weinheim.

Phage display-derived peptides are clinically promising due to their high specificity, selectivity and targeting characteristics. However, functional peptides identified from traditional phage display are composed of naturally occurring L-amino acids (**Figure 4A**). These peptides are prone to degradation by proteases and therefore have a short half-life 40. The chemical reactions in which enzymes degrade peptides are stereoselective, and the rate of reaction is highly correlated with the configuration of the reactant. *In vivo* degrading enzymes can effectively recognize the natural configuration of peptides and make them inactive, which limits the applications of peptide drugs. While D-configuration peptides (enantiomers of the corresponding L-configuration peptides) cannot be degraded by degrading enzymes and are highly stable under physiological conditions. Taking into account this, Kim' groups proposed the concept of mirror image phage display technology, which greatly expanded the application of phage-derived peptides *in vivo* 41.

Mirror image phage display mainly takes advantage of chiral amino acids and, consequently, of the peptides or proteins that they form (**Figure 4B**). Its principle is described as following: biopanning is first carried out against the image of the target molecule (D-target) to obtain an affinity phage display-derived L-ligand, and then the corresponding D-ligand could be chemically synthesized. Depending to the mirror symmetry theory, the D-ligand could specifically bind to the naturally-existing L-target molecule. Willbold's group took the lead in applying mirror phage display in the diagnosis and treatment of AD. A randomized 12-mer peptide phage library was used to select against Aβ_42_ fibrils consisting of all D-amino acid residues confirmed by circular dichroism (CD) assay (**Figure 4C**). The most representative D-enantiomeric peptide named D1 was selected and chemically synthesized. D1 was able to inhibit Aβ aggregation and specifically bind to natural Aβ amyloid fibrils in the brain tissue of AD patients (**Figure 4D**) 42, 43. A series of D1-derived peptides have further demonstrated their applicability for *in vivo* molecular imaging 44, 45. This highlights the great potential of mirror image phage display in the AD diagnostic and treatment.

The mirror image phage display circumvents the striking disadvantages that functional peptides derived from the phage display biopanning are partially worthless for therapeutic and diagnostic applications in living animals or humans. Owing to the remarkable advantages including strong protease resistance, low immunogenicity, and long half-life *in vivo*, the mirror image phage display derived peptides composed of D-amino acids might be more suitable for peptide drug discovery.

### Binding affinity evaluation of phage displayed peptides

Even after multiple rounds of biopanning, the resulting peptide may not be the true affinity binder of the target molecule. Bakhshinejad *et al*. found that they and another research group generated the same phage selection peptide TLHPAAD on completely different targets 46. Further investigation demonstrated that phage clone displaying TLHPAAD peptide bound to polystyrene surface of the solid phase with a significantly higher affinity. Therefore, it is essential to evaluate and compare the affinity of each phage displayed peptide to its target.

Phage enzyme-linked immunosorbent assay (ELISA) is sufficient for rapidly determining the binding affinity between the selected phage clone and the target. In general, a microtiter plate is coated with the target, and each purified phage clone is applied to the plate at various dilutions, followed by the detection with an HRP-labeled anti-M13 antibody, and the final added chromogenic substrate is catalyzed by HRP to produce a colored product and its absorbance at a specific wavelength is recorded. It can be used to quickly determine the relative binding affinity of multiple selected clones in parallel, and to distinguish between genuine target binding and plastic binding 47, 48.

The binding affinity of synthetically generated phage displayed peptide with target can also be measured with various classical affinity methods, such as isothermal titration calorimetry (ITC) 49, 50, surface plasmon resonance (SPR) 51, microscale thermophoresis (MST) 52, and quartz crystal microbalance (QCM) 53,* etc*. The measurement method mentioned above for evaluating biomolecule interactions is capable of quantitatively measuring association constants (*K*_a_) or dissociation constants (*K*_d_) between the peptide and the target molecule. Puhl *et al*. found that the calcium and integrin binding protein 1 (CIB1) affinity peptide screened by the phage peptide library bound to CIB1 with a high nanomolar affinity (*K*_d_ = 29.4 ± 9.5 nM) in the ITC assay 50. In addition, recent advances in electrochemical methods such as cyclic voltammetry (CV) and electrochemical impedance spectroscopy (EIS) were also used to investigate the binding events 54, 55.

## Phage display technology-based AD therapy

### Methods for therapeutic evaluation of peptides

Phage display offers an unprecedented opportunity to improve the therapeutic potential of peptides for AD. Phage display strategy has a clear target and explicit therapeutic mechanism, mainly targeting several major pathological hallmarks of AD, such as deposition of Aβ and tau, disorder of metal ion metabolism. Numerous potential candidate peptides have been discovered through high throughput phage peptide library selection (**Table 1**). In addition to the affinity measurements mentioned above, the current literature explores a range of experimental methods for evaluating the specificity and therapeutic efficiency of phage display derived peptides in the AD application.

Peptide inhibition of AD-associated amyloid aggregation *in vitro* is a prerequisite for their ability to operate in the more complex environment of cells and *in vivo*. Thioflavin T (ThT) fluorescence assay was used to monitor the process of peptide inhibition of amyloid fibrillation in real time, because the fluorescence intensity of ThT positively correlates with the content of the misfolded protein β-sheet structure 56. Similarly, the morphological changes of phage display peptides affecting the aggregation of Aβ peptide or tau protein are usually observed by transmission electron microscopy (TEM) and atomic force microscopy (AFM) 57, 58. Furthermore, CD spectra 59 and two-dimensional nuclear magnetic resonance (2D-NMR) 60 are also complemented with fixed point and *in vitro* detection.

At the cellular level, it mainly evaluates the biocompatibility of phage-derived peptides, whether it can penetrate cell membranes, and recuse toxic factors such as Aβ/tau oligomers and fibers induced cell cytotoxicity 57, 58. The application of functional peptides in animal models of AD represents a more realistic therapeutic efficiency under physiological conditions. The decrease of SPs and NFTs in AD transgenic (Tg) mice are undoubtedly the most important indicators 59, 60. Staining of brain tissue sections (Congo red, ThT, or antibody staining) or western blot analysis of proteins are the classic evaluation measures 35. Besides that, in behavioral neuroscience studies, Morris water maze and nesting construction assays were used to evaluate the effects of peptides on learning, memory and cognitive impairment in AD model animals 62. In general, *in vitro*, cell and model animal exploration of the functionality of phage display peptides is the premise of making them drugs.

### Peptide inhibitors of Aβ aggregation

Among diverse disease-related molecules, accumulation of Aβ has emerged as the primary focus of studies in the pathophysiology of AD 1. The build-up of Aβ into senile plaques contributes to numerous detrimental effects on neurons and other cell types in the brain 63. Therefore, prevention, inhibition and elimination of amyloid deposition in the brain of AD are promising. Aβ fibril formation is a self-assembly process that initiates with a lag phase (oligomer/critical nucleus formation), followed by elongation (oligomer polymerization) and fibril maturation 64-66, and these different Aβ conformations have also been used as selection baits for phage display technology.

In 2006, Orner *et al*. identified several Aβ-affinity peptide ligands through phage display 51. The targets that they screened were Aβ intermediates with two different conformations: monomeric and highly aggregated ones. Interestingly, they found that the peptides identified from the selection for the Aβ monomeric state had little effect on Aβ aggregation, but those peptides selection against the Aβ aggregated state increase the rate of Aβ aggregation. The underlying mechanism was that the peptides might have facilitated the conversion of soluble, toxic Aβ oligomers into insoluble Aβ aggregates to reduce their toxicity. This highlights the ability of phage displayed peptides to recognize distinct conformations of the proteins and produce different affinity ligands. Moreover, SPR-based affinity assay revealed that the endogenous peptide to modulate aggregation correlated with its affinity for the *N*-terminal 10 residues (Aβ_1-10_). Armed with this knowledge, a highly specific Aβ_1-10_ affinity peptide (PYRWQLWWHNWS) was identified by phage display 67. TEM analysis showed the addition of both the selected phages and special synthetic phage displayed peptides can perturb obvious Aβ plaques into bundles of short fibrils. The peptide alleviated Aβ-induced PC12 cell viability, apoptosis and exhibited a protective effect against Aβ-induced learning and memory deficits in rats.

**Figure 5:**
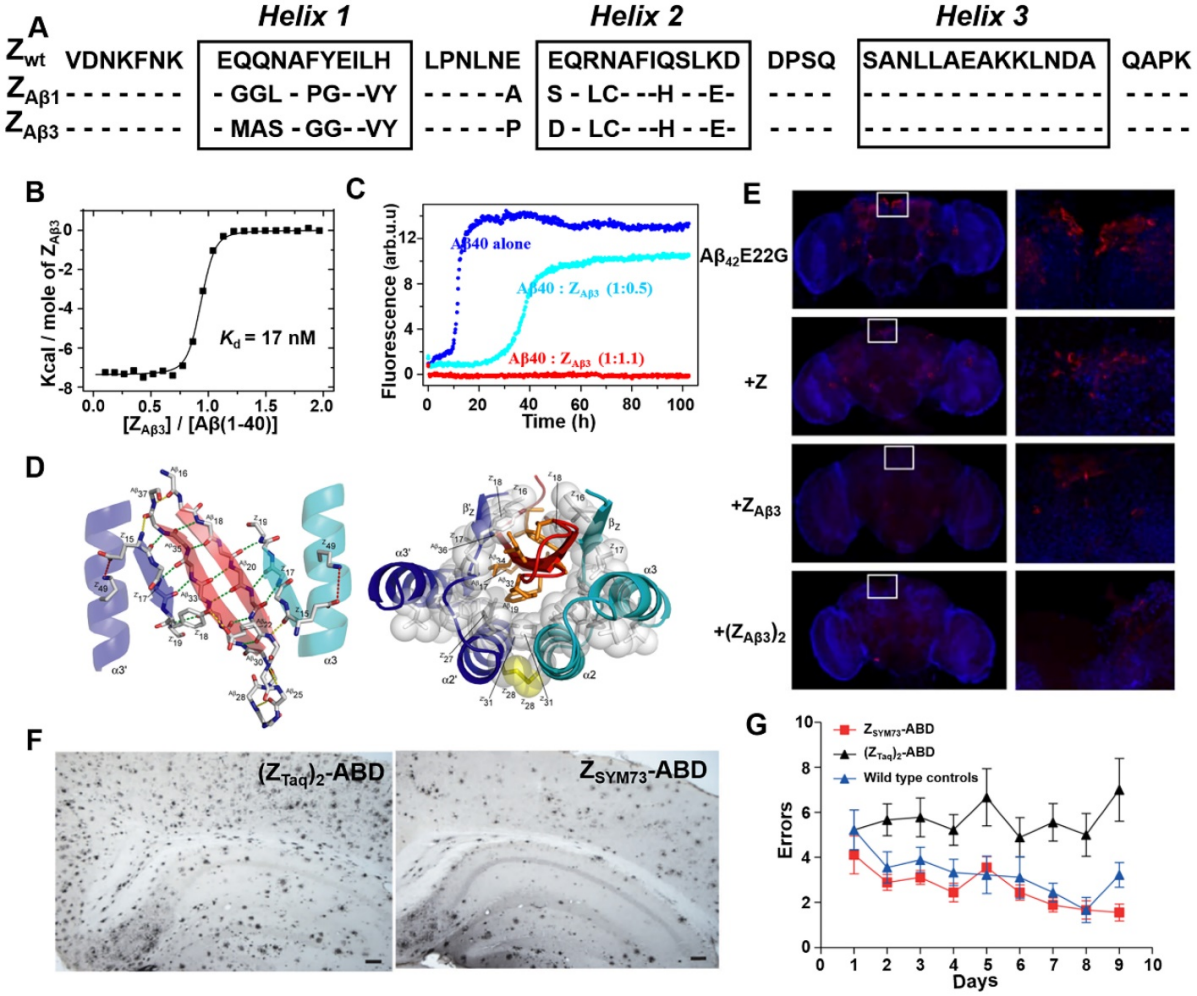
**Phage display-derived affibody targeting Aβ and their functional characterization.** (A) Amino acid sequences of Z_Aβ1_ and Z_Aβ3_ selected against the Aβ peptide. (B) Titration of Z_Aβ3_ dimer into Aβ (1-40) monitored by ITC. (C) Aggregation time course of Aβ (1-40) in the absence (blue) and presence of 0.5 (cyan) or 1.1 (red) molar equivalents of Z_Aβ3_ dimer monitored by ThT fluorescence. (D) Structure of the Z_Aβ3_: Aβ (1-40) complex. (E) Immunofluorescence analysis of intact brains using Aβ-binding antibodies 6E10/4G8 from flies expressing Aβ_42_E22G alone or in combination with Z domain control, Z_Aβ3_ or (Z_Aβ3_)_2_ affibody constructs. Anti-Aβ immunostaining is shown in red, with a nuclear counterstain (TOTO-3) shown in blue. (F) Immunohistochemical images of total amyloid burden using Aβ-binding antibodies 6E10/4G8 on brain sections from Z_SYM73_-ABD or (Z_Taq_)_2_-ABD control-treated APP/PS1 Tg mice. Z_SYM73_-ABD reduced total amounts of amyloid burden in the regions of cortex and hippocampus. (G) Radial arm maze testing of the Z_SYM73_-ABD (red) and (Z_Taq_)_2_-ABD (black) treated AD Tg mice. The Z_SYM73_-ABD treated mice navigated the maze with fewer errors than (Z_Taq_)_2_-ABD group. (B-D) Reprinted with permission from 49. Copyright (2008) National Academy of Sciences, U.S.A. (E) Licensed under a Creative Commons Attribution (CC BY) license. (F, G) Licensed under a Creative Commons Attribution (CC BY) license.

By generating and screening a combinatorial phage protein library based on the 58-amino acid residue staphylococcal protein A-derived Z domain, Grönwall *et al*. developed a series of affibody ligands specific for human Aβ_40_ 68. Among the 16 identified affibody variants, the two most promising affibody variants (Z_Aβ1_ and Z_Aβ3_) (**Figure 5A**) were shown to predominantly bind to Aβ peptides, and enable to capture Aβ peptides from human plasma and serum samples. Affibody molecules in dimer form were constructed to improve the binding ability with Aβ peptides. Further ITC and ThT fluorescence assay indicated dimeric Z_Aβ3_ (abbreviated as (Z_Aβ3_)_2_) bound to the monomeric Aβ_40_ with a high affinity of *K*_d_ = 17 nM (**Figure 5B**) and could inhibit Aβ_40_ fibrillation at stoichiometric concentrations (**Figure 5C**) 49. NMR structural analysis revealed that two Z_Aβ3_ molecules linked by disulfide bond encapsulated the aggregation-prone part of the Aβ peptide, thereby inhibiting Aβ_40_ aggregation (**Figure 5D**). In *in vivo* study using Aβ-Tg fruit fly models, both Z_Aβ3_ and (Z_Aβ3_)_2_ were able to promote the clearance of Aβ from the *Drosophila* brain (**Figure 5E**) 69. In 2015, in order to further improve the affinity between Aβ_40_ and Z_Aβ3_, Lindberg* et al*. rationally improved the molecular structure of Z_Aβ3_ using staphylococcal cell surface display combined with fluorescence-activated cell sorting (FACS) approach 70. The best performing candidate affibody Z_SYM73_ bound Aβ with an approximate *K*_D_ of 340 pM, corresponding to a 50-fold improvement in affinity relative to Z_Aβ3_. In their work published in 2019, Z_SYM73_ and albumin binding domain (ABD) were genetically linked (Z_SYM73_-ABD) to increase Z_SYM73_ *in vivo* half-life. Z_SYM73_^_^ABD treatment reduced amyloid burden in the brains of APP/PS1 Tg mice (**Figure 5F**) and rescued cognitive functions in behavioral tests (**Figure 5G**) 71.

Taking into account the limitation of natural configuration of peptides sensitive to proteases* in vivo* application, Willbold's group conducted a series of work to develop D-peptide inhibitors based on mirror image phage display technology over the last 20 years. The novel and potential candidate D-enantiomeric peptide D3 (RPRTRLHTHRNR) was identified against Aβ_42_ monomeric or small oligomers 72. ThT fluorescence and fluorescence correlation spectroscopy (FCS) assay suggested that D3 could not only inhibit Aβ aggregation, but also redissolve pre-existing Aβ fibrils. D3 treatment can rescue Aβ-induced cytotoxicity in PC12 cells and significantly reduced the amount of inflammation and Aβ plaque load of Tg mice. Furthermore, oral administration of the D3 improved the cognitive performance of both young and old AD Tg mice and yielded a substantial reduction in the amount of amyloid deposits and associated inflammatory response 73, 74. Further pharmacokinetic studies have demonstrated that D3 had high proteolytic stability, effective penetration through the brain and high oral biocompatibility 75. Based on these research, several rational design D3 derivatives, for instance, D3D3 76, RD2 77, RD2D3 78, were also subjected to *in vitro* and *in vivo* investigation. D3D3 is the head-to-tail tandem version of D3, which has a higher affinity with Aβ and a higher efficiency in eliminating Aβ_42_ oligomers. RD2 is a rationally designed reshuffled form of D3 with a C-terminal penta-D-arginine sequence, and has revealed enhanced Aβ oligomer elimination efficacy compared to D3 both *in vitro* and *in vivo* 79. RD2 exhibited a favorable pharmacokinetic properties and had successfully completed clinical phase 1 80. RD2D3 is a head-to-tail tandem peptide derivative composed of D3 and RD2, which exhibits a lower plasma clearance rate and higher bioavailability than D3D3 after intraperitoneal injections.

### Peptide inhibitors of Tau aggregation

Emerging evidence indicates that aggregated, hyperphosphorylated forms of tau may be a primary driver of neurodegeneration in AD 81. In view of the lack of efficacy of amyloid β-targeted therapy for AD so far, interest is growing in tau as a potential alternative target. Tau is a microtubule-associated protein, which plays an important role in stabilizing microtubules and promoting microtubule assembly under physiological conditions. However, tau can be aggregated into straight or paired helical filaments through dimerization and oligomerization reactions, which further combine to form neurotoxic NFTs 82, 83. Two hexapeptide motifs with high β-sheet propensity in the second and third repeat domains of tau, named PHF6* (^273^VQIINK^284^) and PHF6 (^306^VQIVYK^311^), respectively, are two key sequences in tau fibrillization 84. Due to their importance to the aggregation process, the whole repeat domain, PHF6 and PHF6* constitute an interesting target for phage display technology to interfere with tau assembly 60, 85.

**Figure 6:**
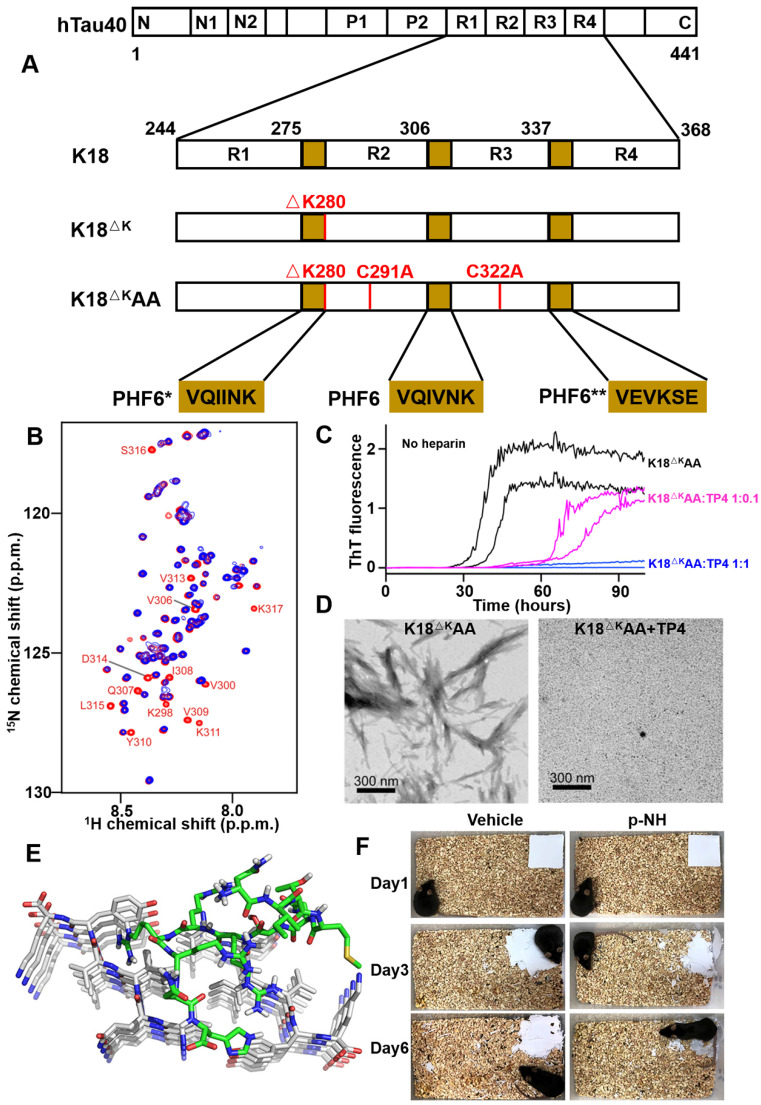
** Phage display derived peptides targeting tau and their applications characterization.** (A) Tau isoform hTau40, Tau constructs K18, K18^ΔK^ and K18^ΔK^AA. hTau40 is the longest human Tau isoform. hTau40 and K18 contain four repeats designated R1 to R4. Deletion and mutations in the constructs K18 and K18^ΔK^AA are indicated in red. The positions of the hexapeptide motifs PHF6, PHF6*, and PHF6** are highlighted in yellow. (B) NMR of the K18-TP4 interaction. ^1^H-^15^N HSQC spectra of K18^ΔK^AA in the absence (red) and presence (blue) of TP4. (C) ThT monitored kinetic curves for K18^ΔK^AA alone (black), K18^ΔK^AA with TP4 atstoichiometric (blue) and substoichiometric (magenta) concentration. (D) TEM of K18^ΔK^AA samples and with addition of TP4. (E) Binding mode of D-enantiomeric peptide p-NH with the PHF6 fibrils. (F) Representative images of nest construction. p-NH treatment rescued the nest construction ability of APP/PS1 Tg mice during 6 consecutive days of observation. (A-D) licensed under a Creative Commons Attribution (CC BY) license. (E, F) Reprinted with permission from 59. Copyright 2020 American Chemical Society.

In 2014, Grüning *et al*. reported an engineered binding protein, β-wrapin TP4 obtained by phage display, using the tau four-repeat construct K18ΔK280 (280 Lysine deletion) as a target (**Figure 6A**) 60. Lysine mutation at site 280 of tau protein is the promoting condition of tau aggregation 86. TP4 bound to K18ΔK280 as well as the longest isoform of human Tau, hTau40, with nanomolar affinity, while did not exhibit an ITC-detectable affinity for Aβ. Additionally, NMR spectroscopy determined that the binding of TP4 to K18 required two Tau hexapeptide motifs: the PHF6 motif and one of the motifs PHF6* or PHF6** (^306^VEVKSE^311^) (**Figure 6B**). ThT fluorescence and TEM confirmed that TP4 effectively inhibited tau aggregation in a concentration-dependent manner (**Figure 6C and 6D**).

Recently, Zhang *et al*. investigated a novel D-enantiomeric peptide p-NH (NITMNSRRRRNH) against D-PHF6 fibrils using mirror image phage display technology 59. p-NH in a D-configuration inhibited PHF6 aggregation in concentration-dependent manner, by comparison, L-p-NH showed no effective fibers inhibition rate. Further molecular modeling study showed that p-NH interacted with PHF6 fibrils mainly through Van der Waals forces and hydrogen bonding to block the addition of another layer of PHF6 and leave them in a blocked anti-aggregation state, thereby effectively inhibiting fibrils growth (**Figure 6E**). Furthermore, p-NH reduced tau hyperphosphorylation and aggregation in both okadaic acid-treated N2a cell model and Tau^P301S^ Tg mice model, and significantly attenuated cognitive behavioral deficits of mice in Morris water maze and nest construction test (**Figure 6F**). The preliminary evaluation of p-NH consisting of all-D amino acids in AD animals demonstrates that it is promising as a clinical candidate drug, and also highlights the enormous potential of mirror image phage display technology in targeted tau therapy.

Funke group developed a series of D peptide therapy strategies based on mirror image phage display technology. PHF6 and PHF6* as targets were screened in two works, respectively, and several D peptides were identified and synthesized 57, 85. Further ThT fluorescence and morphology characterization assays demonstrated that the peptides could inhibit tau constructs and the full tau protein aggregation *in vitro*. However, in-depth cellular, animal-level tests are required to prove the peptide's effectiveness.

Phage display holds enormous potential for developing therapies that target tau, whose pathology is more closely associated with cognitive and functional decline than Aβ. Except for tau aggregation, numerous post-translational modifications (PTM) of tau have long been taken into account to affect protein function and lead to neurodegenerative diseases including AD. This includes the widely known phosphorylation modification, as well as glycosylation, acetylation, methylation and ubiquitination 87, 88. In recent years, more and more new PTM sites of tau highly associated with disease progression have been identified, such as phosphorylation-tau 217 89, 90, ubiquitination-tau 311 88, and acetylation-tau 280 91. Therefore, there is an urgent need to develop inhibitors targeting these PTM sites, and the phage display technology platform will undoubtedly provide sturdy support.

### Peptide chelators regulate metal-triggered AD

Multiple lines of evidence suggest that the homeostasis of transition metal ions plays a pathogenic role in AD 92. Evidence of a link between AD and metal disorders has been supported by postmortem analysis of amyloid plaques, which showed copper, iron, and zinc accumulation, respectively, to be 5.7, 2.8, and 3.1 times higher than levels observed in normal brains 93. The binding of metal ions to Aβ peptide leads to Aβ fibrillation, oxidative stress, and synaptic dysfunction 94. Metal chelators are considered to be potential therapeutic agents for inhibiting and regulating metal-triggered Aβ aggregation in AD.

**Figure 7:**
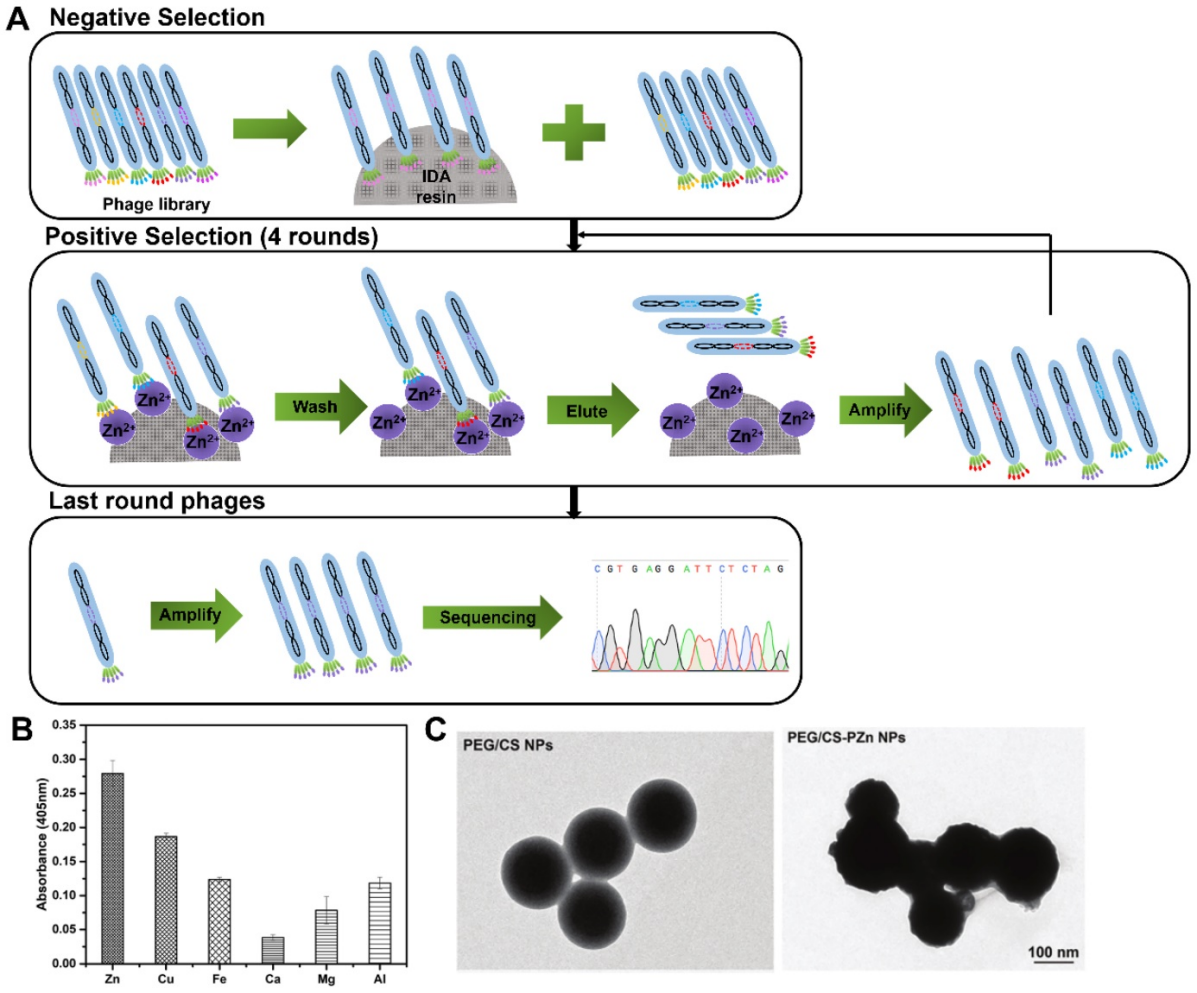
** Phage display selection Zn-binding peptides.** (A) Schematic diagram illustrating the whole biopanning procedure against Zn (II). Zn(II)-binding heptapeptides were identified through one round of reverse selection against blank resin of IDA and four rounds of selection against immobilized Zn(II) resin. (B) The binding affinity of PZn peptide toward different metals. (C) TEM micrographs of PEG/CS NPs and PEG/CS-PZn NPs. Reprinted with permission from 62.

In 2019, Zhang and co-workers first reported an Zn(II)-binding peptide based on phage display for AD application 62. The biopanning was conducted one round of negative selection against blank iminodiacetic acid (IDA) resin and four rounds of positive selection against IDA-Zn (II) (**Figure 7A**), and a total of 15 sets of Zn(II)-binding peptides with relatively high histidine content were identified. The most potential candidate peptide PZn with the sequence of HMQTNHH, had the highest affinity and specificity toward Zn(II), but not toward other metal ions, such as Cu(II), Fe(III), Ca(II), Mg(II) and Al (III) (**Figure 7B**). Polyethylene glycol (PEG)-modified chitosan nanoparticles (NPs) were loaded with the PZn (abbreviated to PEG/PZn-CS NPs) to maintain PZn peptide stability and biocompatibility (**Figure 7C**). PEG/PZn-CS NPs had a certain sustained release property (73% of PZn released at 72 h) and exhibited anti-oxidative and anti-apoptotic responses, and rescue cytotoxicity in N2a-sw cells induced by Zn(II). PEG/CS-PZn NP treatment reduced Aβ burden and ameliorated cognition and memory impairments in APP/PS1 Tg mouse. Further mechanistic analysis demonstrated NPs might alter the redistribution of zinc rather than decrease zinc levels to inhibit Zn(II)-mediated Aβ deposition in the mice brain.

A very recent paper using the similar strategy reported a novel Cu-binding peptide using the phage display technique to screen for a potential inhibitor for Cu(II)-induced Aβ peptide aggregation 58. A heptapeptide sequence of SAQIAPH (PCu) as a potential Cu(II)-binding peptide was identified, which could remarkably inhibit Cu(II)-induced Aβ aggregation and mediates the cellular toxicity. Further investigations have revealed that the PCu peptide could reduce the Aβ levels by targeting the Cu(II)-induced beta-site amyloid precursor protein cleaving enzyme 1 (BACE1) expression and improve the Cu(II)-induced cell oxidative damage. Inductively coupled plasma mass spectrometry (ICP-MS) further demonstrated the ability of PCu to chelate the intracellular level of copper to inhibit the production of Aβ aggregates.

The AD metal ion hypothesis suggests that high concentrations of metal ions (*e.g.*, zinc and copper) play important roles in promoting Aβ deposition and neurotoxicity. Importantly, the imbalance of metal homeostasis in the brain ultimately leads to cognitive decline in different experimental models. Phage display technology provides new ideas for the prevention or treatment of AD by developing novel biocompatible chelating agents targeting metal ions. As more functions of metal ions in the brain are discovered, various other metal ions besides Zn and Cu ions will also become the bait for selection. In addition, in the context of metal chelation therapy, it seems that it is not enough to consider the effect of a single metal element on AD, and how to ensure that the chelating agent only chelates excess metal ions without affecting their normal functions *in vivo* will be an important research direction for the treatment of AD in the future.

### Peptide inhibitors targeting other AD-associated molecules

Receptor for advanced glycation end products (RAGE), a multi-ligand receptor, serves as a cell-surface receptor for Aβ and mediates amyloid β-induced perturbations in AD. Cai *et al*. obtained a RAGE antagonist peptide RP1 (APDTKTQ) screened from a phage display peptide library 95. RP-1 blocked the Aβ-RAGE interaction, inhibited Aβ-induced cellular stress by activation of the PI3K/AKT pathway in SH-SY5Y cells. Furthermore, intranasal administration of RP-1 in APP/PSI Tg mice for 4 months showed improvement in memory impairment and reduction in Aβ plaques 96.

In non-amyloidogenic pathway, BACE1 plays an important role in the sequential cleavage of amyloid precursor protein amyloid precursor protein to form Aβ. A BACE1 specific affinity peptide was successfully identified by phage display 97. The peptide had the ability to prevent BACE1 activity and reduced the amounts of Aβ_40_ and Aβ_42_ production induced in SH-SY5Y cells treated by H_2_O_2_.

## Brain drug delivery in AD: Phage display derived-peptides penetrate BBB

Among the various challenges in drug development, central nervous system (CNS) drugs are more likely to fail than non-CNS drugs due to the existence of blood-brain barrier (BBB), the most tightly regulated interface in the brain. BBB represents a formidable hurdle to deliver drug across the endothelial cell lining into the brain in the treatment of CNS disease, including AD. Therefore, the development of functional carriers that can deliver peptides or other drugs to the brain, thus ensuring drug efficiency and reducing drug adverse effects, is a prerequisite for clinical success in the treatment of AD. The recent advances in the development of brain targeted ligands by phage display technology indicate that it is a reliable and valuable tool. We review several BBB penetrating peptides developed in recent years by phage display *in vitro* and *in vivo* selection strategies and their applications in AD targeting and therapy.

### *In vitro* selection

**Figure 8:**
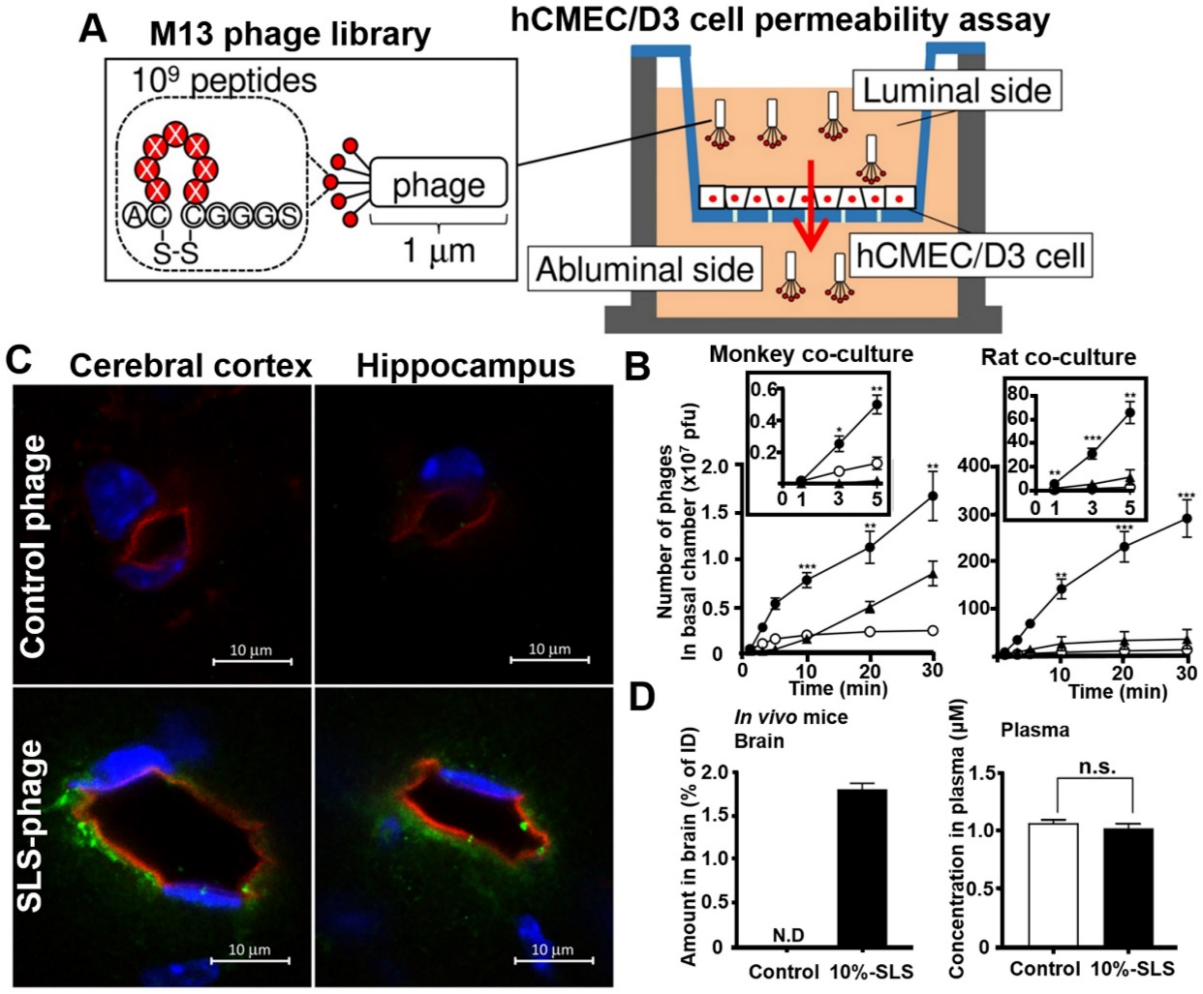
** Phage selection on cell models for BBB penetrating peptides.** (A) Phage library selection for BBB-permeable cyclic heptapeptides with the hCMEC/D3 cell permeability assay. Phage library was added on the apical side, and phages were collected from the basal side to penetrate through the hCMEC/D3 cell monolayer. (B) Permeability assay with identified phage clones that crossed the *in vitro* monkey and rat co-culture BBB models. The black box represents an enlarged view of the number of phage penetrations to the basal side in the first 5 min. Closed circle, SLS phage; closed triangle, NTG phage; open circle, control phage. (C) Distribution of SLS-phages in the mouse brain after intravenous administration (phage, green; blood vessel, red; nuclei, blue). (D) Contents of 10% SLS-liposomes and control liposome in brain and plasma after intravenous administration into the mice. Reprinted with permission from 98. Copyright 2020 ELSEVIER.

Phage display *in vitro* selection is a simple, convenient, and more direct strategy for obtaining brain-penetrating peptides. Its targets are usually a typical BBB cell models or cell receptors that are highly expressed on BBB. Yamaguchi *et al*. applied human cerebral microvascular endothelial hCMEC/D3 cell permeability assay for identifying multiple BBB permeable peptides using *in vitro* phage selection 98. The cyclic heptapeptide phage library was added on the luminal side of the hCMEC/D3 cell monolayer, and the phages capable of penetrating the cell monolayer were recovered on the abluminal side after a short period of permeation (1-3 minutes in the third round) for a total of three rounds of penetration assays (**Figure 8A**). One of the recovered phage was named SLS phage, and the displayed cyclic heptapeptide sequence was C-SLSHSPQ-C (SLS peptide). SLS significantly promoted M13 phages penetration in monkey and rat BBB co-culture model (**Figure 8B**). Intravenous administration of mouse SLS-phages showed that cyclic SLS peptides distributed around the brain microvessels in the cerebral cortex and hippocampus (**Figure 8C**). The cyclic SLS peptide also promoted the non-permeable compound 5/6-carboxyfluorescein (FAM), and liposome permeation across the mouse BBB (**Figure 8D**), thus providing an effective tool for conjugated drug delivery to the brain for the treatment of AD.

In addition to the BBB cell model, the receptors in the brain endothelial cells were also used as bait to obtain some peptide ligands that could explicitly recognize these receptors, so that the biotherapeutics agent were able to be carried into BBB through receptor-mediated transport (RMT). Several RMT transporting systems have been described, for instance, transferrin receptor 99, 100, low-density lipoprotein receptor (LDLR) 101, *etc*. André *et al*. employed phage dodecapeptide library targeted extracellular domain of LDLR (ED-LDLR) 102. The LDLR-targeted peptide LRPep2 (HPWCCGLRLDLR) interacted with the interface between R4 and the β-propeller (βP) domain of ED-LDLR. *In vivo* fluorescence evaluation demonstrating a stronger fluorescence was observed in the area of the brain of mice after injection with LRPep2.

### *In vivo* selection

Despite using BBB cell models or cell membrane receptors is a direct and convenient method for biopanning, it is well known that the *in vitro* condition can be quite different from the complex brain environment *in vivo*. Phage display *in vivo* has been proven to be very effective in selecting phages with high organ specificity after systemic injection 38, and several BBB penetrating peptides from *in vivo* selection have been identified and applied to targeted delivery of AD drugs 61, 103.

Li *et al*. applied *in vivo* selection approach to inject phage dodecapeptide library to mice through tail vein, and phages were recovered from brain after 24 h 38. The phage clone 12-2 displaying peptide TGN (TGNYKALHPHNG) showed a superiority transport efficiency into the brain than other organs. The TGN conjugated NPs resulted in higher cellular uptake by bEnd.3 cells and brain uptake in nude mice after vein administration. Based on this knowledge, Zhang and his colleagues constructed a dual-targeted delivery NP capable of both brain targeting and further Aβ targeting 103. The nanoparticles were constructed by surface conjugation of TGN and another Aβ_42_ affinity peptide QSH, also screened by phage display. The dual-functional targeted NP system achieved enhanced and precisely targeted delivery to amyloid plaques in the brains of AD model mice.

**Figure 9:**
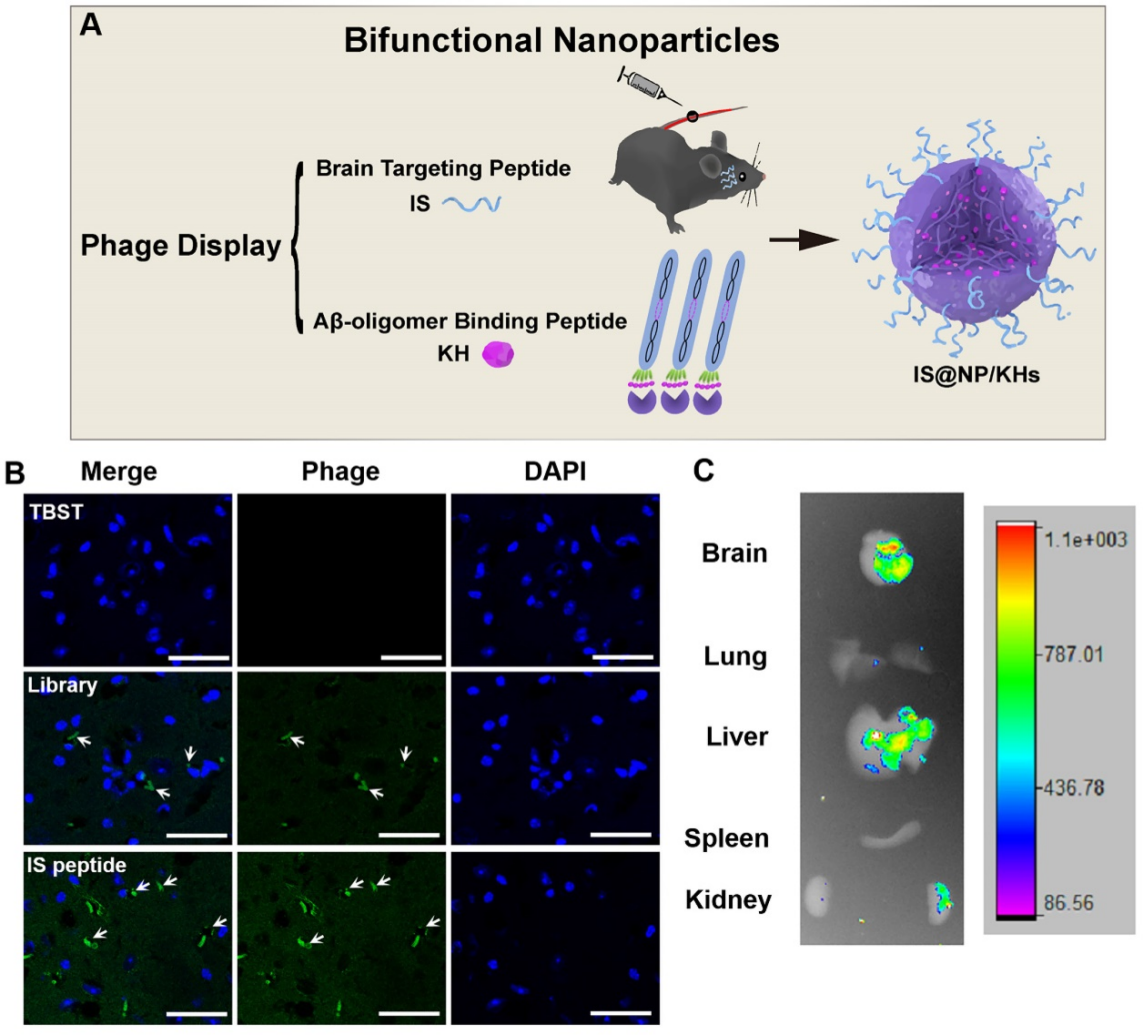
** Preparation of bifunctional nanoparticles and evaluation of the peptide brain targeting function.** (A) Schematic illustration of the preparation of IS@NP/KHs. Both Aβ_42_ oligomer affinity peptide KH and brain targeting peptide IS were identified from phage display. Subsequently, IS peptide and KH peptide were assembled into bifunctional NPs (IS@NP/KHs). (B) Phage immunofluorescence staining of the brain sections of APP/PS1 mice. TBST, phage library, and phage clone encoding IS peptide were injected into APP/PS1 mice through the tail vein, respectively, and anti-M13 antibody immunofluorescence staining was performed on the brain sections (phage, green; nuclei, blue). (C) *Ex vivo* imaging of FITC-IS in main organs. APP/PS1 mice were injected with FITC-labeled IS via a vein tail route. Optical images showing the brain, lung, liver, spleen, and kidney of FITC-IS administered mice after 2 h. Reprinted with permission from 59. Copyright 2021 American Chemical Society.

In 2021, Zhang *et al*. combined phage *in vitro* and *in vivo* selection strategy to develop a novel bifunctional NP for the treatment of AD (**Figure 9A**) 61. They obtained an Aβ oligomer affinity peptide KH (KSILRTSIRHTH) as an Aβ aggregation inhibitor by phage *in vitro* selection, and a brain targeted peptide IS (ITPTRKS) by phage *in vivo* selection for brain targeted delivery. The IS encoding phage clone showed higher brain targeting efficiency (**Figure 9B**) and *ex vivo* imaging of small animals demonstrated that fluorescein isothiocyanate (FITC)-labeled IS peptide could enter into the mice brain after vein injection (**Figure 9C**). Furthermore, KH peptides were encapsulated in the internal space of chitosan crosslinked NPs, and the IS peptides were appended to the chitosan surface to form bifunctional NPs (IS@NP/KHs). IS@NP/KH could effectively inhibit Aβ deposition in the brain of APP/PS1 Tg mice and significantly reduce their cognitive and behavioral deficits.

## Phage display technology for AD early diagnosis

To facilitate clinical trials of disease-modifying therapies for AD, early detection and diagnosis at the early stage of AD are necessary and urgent for primary care settings. However, clinically data-based diagnosis of AD is currently supported by a suite of cerebrospinal fluid (CSF), positron emission tomography (PET), and magnetic resonance imaging (MRI), which are available only in specialized clinical settings and obtained through expensive and highly invasive techniques 104, 105. The utilization of phage display to identify accurate, low cost and less invasive AD specific biomarkers is attracting attention.

Apolipoprotein E (ApoE) serving as a lipid-binding protein has been used an early biomarker in clinical diagnosis due to its strong association with AD 106. In 2017, Ren *et al*. reported a colorimetric immunosensor for point-of-care detection of ApoE 107. After bactrian camel was immunized with ApoE, nano antibody cDNA was extracted and constructed, and inserted into the phage genome, the phage display immune nano antibody library was successfully generated. Several anti-ApoE nanobodies were identified after three rounds of phage display biopanning. The two potential candidate nanobodies Nb05 and Nb40 presented high affinities with ApoE (*K*_D_ of 3.40 × 10^-9^ and 8.14 × 10^-10^ M, respectively) in SPR assay, and were subsequently assembled to fabricate an ApoE immunosensor. The ApoE immunosensor constructed based on Nb05 and Nb40 exhibited high precision and accuracy in real sample analysis and have potential application in the clinical diagnosis and real time monitoring for AD.

**Figure 10:**
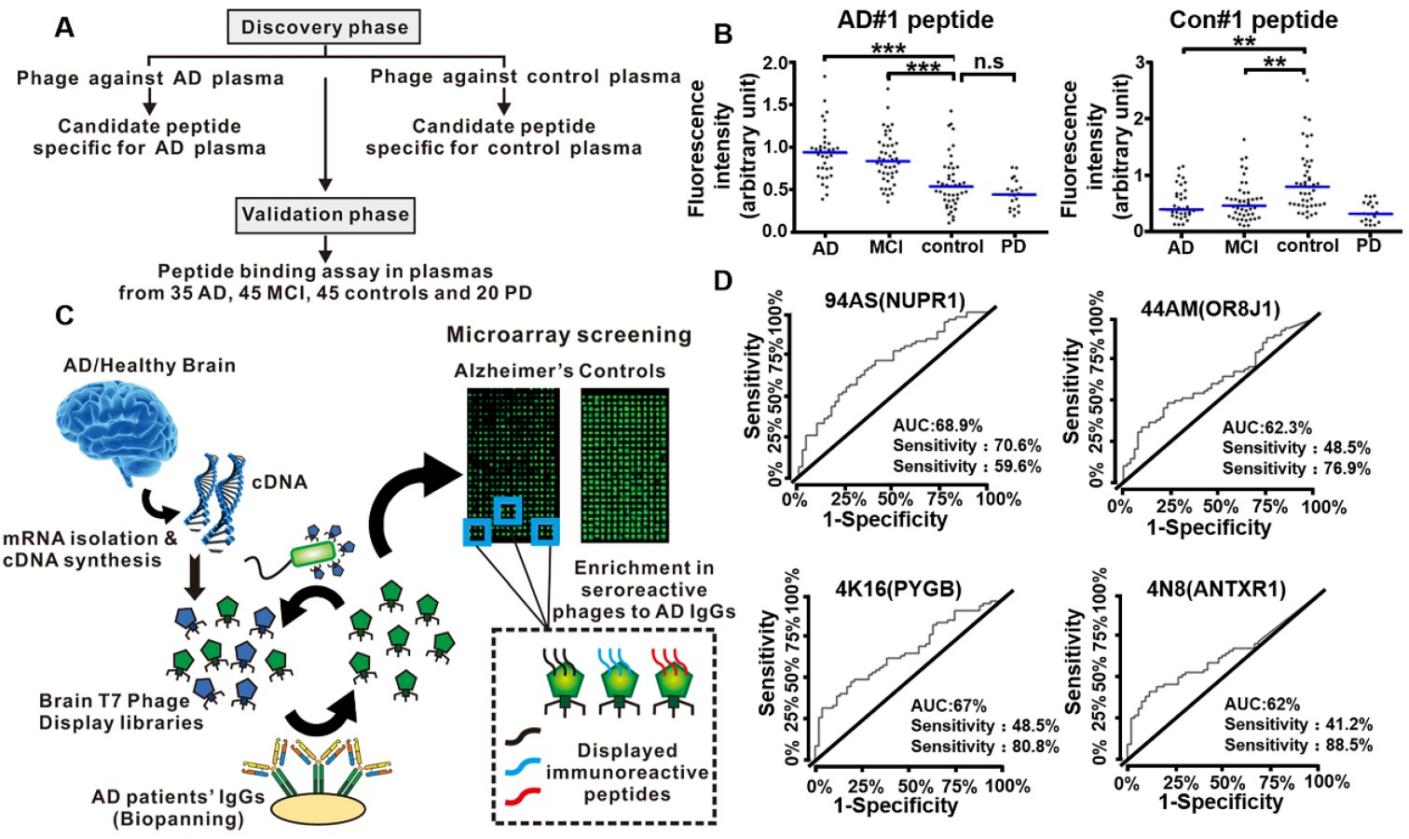
** Phage display technology for early detection of AD.** (A) Diagram showing the discovery phase and validation phase of the entire study. (B) Validation of phage-derived peptides in independent set. Binding of AD#1 peptide and Con#1 peptide in the plasma samples from AD (n = 35), MCI (n = 45), controls (n = 45) and PD patients (n = 20). (C) Workflow for the identification of specific targets of autoantibodies in AD patients' sera using phage microarrays. Two T7 phage libraries displaying cDNA from healthy and AD brain tissues were used to screen against serum IgG from AD patients. The bound phages were isolated, amplified, and transferred to a plate for microarray printing, and further validated with new serum samples from AD patients and healthy individuals. Peptides displayed on the most immunoreactive phages against serum samples from AD patients were cloned and expressed to determine their AD diagnostic ability. (D) Performance of the 94AS, 44AM, 4N8, and 4K16 displayed peptides for AD diagnosis. ROC curves obtained from the analysis of the four peptides showing a higher statistically significant immunoreactivity by 68 serum samples from AD patients than the 52 from healthy controls sera. (A, B) Reprinted with permission from 108. Copyright 2019 ELSEVIER. (C, D) Reprinted with permission from 104. Copyright 2019 American Chemical Society.

In 2019, Chen *et al*. conducted a peptide binding assay to select functional peptide for the early diagnosis of AD 108. The entire study they designed consisted of a discovery phase and a validation phase (**Figure 10A**). The discovery phase was intended to use phage display to screen candidate peptides against plasmas from AD patients and normal healthy people, respectively. Further investigation with ELISA and peptide competition binding assay indicated AD#1 peptide (HMRQGMA) had high and specific affinity to AD plasma, while Con#1 peptide (DGARHGR) specifically bound to healthy people plasma. In the validation phase, an additional independent set of 35 AD patients, 45 mild cognitive impairment (MCI) patients, 45 healthy people as controls, and 20 Parkinson's disease patients were included. They proved that AD#1 could distinguish AD from healthy people and Con#1 peptide could specifically detect the controls through the receiver operating characteristic curve analysis (**Figure 10B**). The combination of these two peptides largely improved the diagnostic performance, which may provide a new blood biomarker test for the early accurate diagnosis of AD.

Autoantibodies and their correspondence target proteins have become promising diagnostic tools for blood-based biomarkers due to their high accessibility, stability and specificity. AD is also considered as an autoimmune disease 109. The autoantibodies (predominantly the IgG class) produced by AD patients could be triggered by AD specific target proteins 110. Phage display integrated protein microarrays were used to screen AD autoantibodies as blood biomarkers for early diagnosis 104. Segundo-Acosta *et al*. used T7 phage libraries displaying AD brain tissue mRNA to identify phages against serum purified IgGs from AD patients (**Figure 10C**). Subsequently, those phages displaying the specific target protein corresponding to AD autoantibodies were extracted and printed on the microarrays for further validation with new serum samples from AD patients and healthy people. Among the thirteen proteins with high immunogenicity with AD serum samples, four proteins, anthrax toxin receptor 1 (ANTXR1), nuclear protein 1 (NUPR1), glycogen phosphorylase (PYGB), and olfactory receptor 8J1 (OR8J1), were identified and showed abilities to distinguish AD patients from the healthy people using a fine-tuned luminescence beads immunoassay, thus being added to the blood-based criteria for AD biomarkers (**Figure 10D**). In 2020, Plano *et al*. performed a double binding phage display selection to identify new conformation antigens of Aβ_1-42_ by alternating biopanning with AD serum and monoclonal antibody YPF19 (chosen as like Aβ-amyloid structure protein), which can be used as novel diagnostic biomarkers for the diagnosis of AD patients 111. Compared with wild-type phage M13, the selected recombinant phage clone 12III1 showed a greater ability to prevent amyloid aggregation and promote disaggregation of preformed fibrils 112. The phage clone 12III1 was implemented to detect the presence of specific antibodies in AD serum in a preliminary explorative study.

In addition to the development of novel biomarkers for *in vitro* diagnosis of AD, phage display-derived peptides have also been used as amyloid plaques-specific contrast agents to achieve *in vivo* molecular imaging. Larbanoix *et al*. identified several Aβ_42_ affinity peptides using a disulfide constrained heptapeptide phage library 34. Two biotinylated peptides, PHOb (C-FRHMTEQ-C) and PHIb (C-IPLPFYN-C), were selected and synthesized due to their picomolar affinity for Aβ_42_ in the ELISA assay. Further immunohistochemistry and preliminary MRI *in vivo* assay on Tg mice model of AD demonstrated that the peptides were able to specifically bind to amyloid plaques and could be act as excellent contrast agents. In their another work published in 2011 35, they designed another linear hexapeptide phage display library based on Aβ_1-42_ amino acid sequence and selected for the aggregation of amyloid Aβ_42_ as a target. The two best peptides, pep1 (LIAIMA) and pep2 (IFALMG), had been verified to have specifically interaction with amyloid plaques in AD Tg mice brain sections. These peptides showed no toxicity in cell culture and were able to inhibit Aβ_42_ aggregation.

The poor prognosis of AD is in large part due to the typically delayed diagnosis of the disease. Early, accurate, and biomarker-based diagnosis of AD may become more important when disease-modifying therapies become available. Blood-based early diagnosis of AD represents a less expensive, less invasive, more manageable test that is already being implemented in the primary care system. Phage display technology was used to screen functional peptides or antibodies from the blood of AD patients and healthy people, respectively, and to develop blood biomarker detection test based on these phage-derived peptides, providing new insights for the early diagnosis of AD. In addition, new biomarkers for AD diagnosis, such as P-tau181 113 and P-tau 217 90, have been emerging in recent years. The application of phage display technology to develop high affinity and highly sensitive probes, which could be combined with various biosensors to achieve rapid blood detection, will show immense potential.

## Challenges of phage display strategies for AD applications

The utilization of phage display to screen functional peptides has been appealing in overcoming the unmet challenges for AD applications. Peptide-based biotherapeutics are clinically promising due to their several robust characteristics, for examples, small size, low cost, easily manufacturing process, consistent reproducibility, highly specific, and low immunogenicity. However, there are some obstacles that still need to be overcome to accelerate the clinical application of peptide in diagnosis, treatment, and target drug delivery of AD.

**Figure 11:**
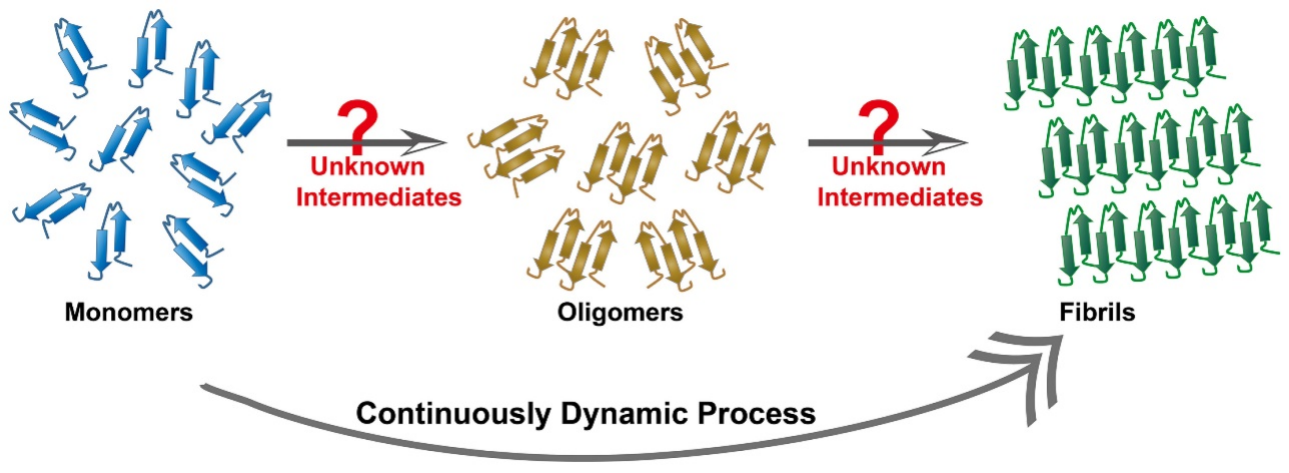
** Continuously dynamic process of proteins aggregation.** Protein fibril formation is a continuously dynamic process, where the process initiates with a lag phase followed by elongation and fibril maturation. Some unknown intermediates are likely to appear during the aggregation process.

Target biomolecules are important and indispensable parameters for phage biopanning. The Aβ cascade hypothesis and the tau protein hypothesis are widely accepted theories to interpret AD pathology. They represent the two dominant target objects in the AD phage selection. In the past two decades, tremendous contribution in distinguishing protein conformation has facilitated the understanding of the process of amyloid aggregation. Therefore, the target molecules by the phage selection in AD is incline to select single and clear objects, such as Aβ oligomers 61, Aβ protofibrils 114, and tau fibers 59. By comparison, intermediate conformation of these proteins, such as transition states from monomers to oligomers, or from oligomers to fibers, are found to have stronger neurotoxicity but these intermediates are dynamic and unstable, being too difficult to be identified (**Figure 11**). In addition, a multitude of currently unknown three-dimensional structures are likely to appear during the aggregation process. Recent research based on cryo-electron microscopy technology has achieved high-resolution structural analysis of Aβ oligomers 115 and tau protein 116. Therefore, the discovery and clarification of the unknown three-dimensional structure and function of more misfolded proteins will be an essential task. Meanwhile, new methods that are capable of freezing or fixing these protein structures need to be strengthened in order to achieve accurate phage selection and targeted therapy.

It is also noteworthy that some synthetic peptides generated by phage display exhibit variable and weak function in treatment or brain delivery targeting. Potential difference between the phage microenvironment and practical physiological condition in the peptide configuration may account for these discrepancies. Moreover, chemically synthetic peptides may not maintain the original configuration displayed at the bacteriophage tip, which confer the target recognition and specificity. Similarly, multiple copies of the exogenous displayed peptide on the phage result in strong binding to the target during the selection process, but only a single synthetic peptide might not be strong enough. A typical example is GYR peptide targeting brain capillary endothelial cell showed weak target engagement on liposome surfaces. Instead, GYR self-assembled into supramolecular core-shell nanoparticles and nanofibers, rather than in its monomeric state, resulting in significantly improved target recognition and binding performance 117. Therefore, sequence analysis and structure prediction of the phage-derived peptides, as well as exploring whether supramolecular assembly of peptides occurs, need to be considered carefully. Cyclic peptides may be a better choice as BBB penetrate peptides than their linear counterparts due to conformational rigidity and structural controllability.

False positives and omitted screen represent additional challenges in the phage display. Considering that phages bind to plastic substrates or blocking agents (such as bovine albumin solution) 46, as well as M13 phages possess negative charge preferentially bound to positive charge vectors 47, a large number of phages have been occupied by nonspecific adsorption prior to the positive selection. Additionally, amplification is necessary for the enrichment of eluted phage after each round of selection, but increases the risk of introducing biological bias and collapse of library diversity. For instances, some phages are not amplified preferentially due to poor infectivity, and wild-type phages introduced due to contamination are preferentially amplified. Recent advances in next generation sequencing (NGS) make it possible to address these concerns. NGS allows for the characterization of more than 10^6^ reads in a single run so that repeated peptide sequences can be identified in the earlier round of biopanning 118. Deep sequencing can also be used prior to the start of the experiment and after each round of biopanning, thereby reducing the risk of identifying false positive phages.

## Concluding remarks and future perspectives

AD research is right on a critical juncture. Despite scientists have made significant progress in understanding the pathobiology of AD, no effective disease-modifying therapy has been identified so far. Phage display strategies have provided an unprecedented opportunity for the treatment of AD. The unique advantages of phage display address the challenges of low targeting and low specificity in drug discovery and biomarkers imaging, and provides strong support for the early diagnosis of AD asymptomatic period, targeted drug delivery and precision medicine. Although applications of phage display technology in cancer and tumors have been extensively reported in a large number of current literature 119, 120, and some phage display derived peptides or antibodies for cancer treatment have been approved by the FDA 121, 122, there have been relatively limited applications and development of the phage display-derived peptides in AD and other neurodegenerative diseases.

In this review, we summarize various peptides screened from the phage display technology for the treatment, early detection and diagnosis, and brain targeted drug delivery of AD, with an aim to broaden the application of phage display technology in the neurodegenerative diseases. Further studies should focus on following issues to facilitate the transition from basic researches to clinical application: (i) In-depth phase display selection toward the AD pathological intermediates, particularly those with definite conformation. (ii) Optimization of biopanning process by introducing NGS. (iii) Self-assembly test of synthetic peptides to clarify the potential relationship between the assembled structure and bio-function. (iv) Discovery of new targets for AD therapy, such as tau phosphorylation and glycosylation. (v) Macrocyclic peptides or unnatural peptides composed by D-amino acids could be put in place to improve the serum stability of the peptides.

Several FDA-approved cancer drugs (*e.g.*, avelumab, moxetumomab pasudodox) have demonstrated the value of phage display as an established and reliable drug discovery platform and hold great promise for exploring the AD field. Ongoing in-depth investigation using the phage display technology will be essential to make key discoveries that will eventually reveal novel AD therapeutic approaches. The phage display technology strategy is now offering new tools and insights to advance our understanding of AD and ultimately bring new hope to AD patients.

## Figures and Tables

**Table 1:** Specific binding peptides screened via phage display technology for AD therapy

Name	Target^a^	Sequence details^a,b^	Ref.
IQ	Aβ_40_	IQTTWSR	123
cSP	Aβ_40_	CSPPLRFFC	124
Z_Aβ3_	Aβ_40_	VDNKFNKEMASAGGEIVYLPNL	68
		NPDQLCAFIHSLHDDPSQSANL	
		LAEAKKLNDAQAPK	
XD4	Aβ_42_	PIKTLPM	125
ZW1	Aβ_42_	SMSARQL	126
**_**	Aβ_1-10_	PYRWQLWWHNWS	127
**_**	Aβ monomer	Ac-FYLKVQSLHHHH-NH_2_	51
		Ac-GRDKLVFFHHHH-NH_2_	
**_**	Aβ fibril	Ac-NYSKMIFSHHHH-NH_2_	
		Ac-HNHKLVFFHHQH-NH_2_	
**_**	Aβ and Zn^2+^	Ac-DFRKLLLSGQSQ-NH_2_	
_	Aβ_42_ oligomer	RGPRGRV	128
ANA 1	Aβ_42_ oligomer	TNPNRRNRTPQMLKR	33
GN	Aβ_42_ fibril	GNLLTLD	129
Mosd1	*^D^*Aβ_42_ monomer	*^D^*YSYLTSYHMWVR	130
D3	*^D^*Aβ_42_ monomer/oligomer	*^D^*RPRTRLHTHRNR	72
D1	*^D^*Aβ_42_ fibril	*^D^*QSHYRHISPAQV	43
TP4	K18△K280	VDNKFNKEMASAGGEMASGPN	60
		LNPDQLCALVHSLHDDPSQSANL	
		MAEAKKLSDAQAPK	
MMD3	*^D^*PHF6* fibril	*^D^*DPLKARHTSVWY	57
p-NH	*^D^*PHF6 fibril	*^D^*NITMNSRRRRNH	59
APT	*^D^*PHF6 fibril	*^D^*APTLLRLHSLGA	85
TL28		*^D^*TTSLQMRLYYPP	
TD28		*^D^*TTSLQMRLYYPP	
TD28rev		*^D^*PPYYLRMQLSTT	
PZn	Zn (II)	HMQTNHH	62
PCu	Cu (II)	SAQIAPH	58
RP-1	RAGE	APDTKTQ	95
PAP11	PirB	PFRLQLS	131
	BACE1	Undisclosed	97

a- D denotes D-amino acids; b- peptide C-terminal and N-terminal modifications are as indicated, where not shown C-terminal and N-terminal peptides are not capped/modified.
